# Effects of prior deployments and perceived resilience on anger trajectories of combat-deployed soldiers

**DOI:** 10.1017/S0033291721003779

**Published:** 2023-04

**Authors:** Laura Campbell-Sills, Jason D. Kautz, Karmel W. Choi, James A. Naifeh, Pablo A. Aliaga, Sonia Jain, Xiaoying Sun, Ronald C. Kessler, Murray B. Stein, Robert J. Ursano, Paul D. Bliese

**Affiliations:** 1 Department of Psychiatry, University of California San Diego, La Jolla, CA, USA; 2 Department of Organizations, Strategy, and International Management, University of Texas at Dallas, Dallas, TX, USA; 3 Department of Psychiatry, Massachusetts General Hospital, Boston, MA, USA; 4 Department of Epidemiology, Harvard T.H. Chan School of Public Health, Boston, MA, USA; 5 Psychiatric and Neurodevelopmental Genetics Unit, Center for Genomic Medicine, Massachusetts General Hospital, Boston, MA, USA; 6 Stanley Center for Psychiatric Research, Broad Institute, Boston, MA, USA; 7 Department of Psychiatry, Center for the Study of Traumatic Stress, Uniformed Services University of the Health Sciences, Bethesda, MD, USA; 8 Henry M. Jackson Foundation for the Advancement of Military Medicine, Bethesda, MD, USA; 9 Biostatistics Research Center, Herbert Wertheim School of Public Health and Human Longevity Science, University of California San Diego, La Jolla, CA, USA; 10 Department of Health Care Policy, Harvard Medical School, Boston, MA, USA; 11 VA San Diego Healthcare System, San Diego, CA, USA; 12 Herbert Wertheim School of Public Health and Human Longevity Science, University of California San Diego, La Jolla, CA, USA; 13 Department of Management, Darla Moore School of Business, University of South Carolina, Columbia, SC, USA

**Keywords:** Anger, military deployment, resilience, military personnel, mixed-effect growth models

## Abstract

**Background:**

Problematic anger is frequently reported by soldiers who have deployed to combat zones. However, evidence is lacking with respect to how anger changes over a deployment cycle, and which factors prospectively influence change in anger among combat-deployed soldiers.

**Methods:**

Reports of problematic anger were obtained from 7298 US Army soldiers who deployed to Afghanistan in 2012. A series of mixed-effects growth models estimated linear trajectories of anger over a period of 1–2 months before deployment to 9 months post-deployment, and evaluated the effects of pre-deployment factors (prior deployments and perceived resilience) on average levels and growth of problematic anger.

**Results:**

A model with random intercepts and slopes provided the best fit, indicating heterogeneity in soldiers' levels and trajectories of anger. First-time deployers reported the lowest anger overall, but the most growth in anger over time. Soldiers with multiple prior deployments displayed the highest anger overall, which remained relatively stable over time. Higher pre-deployment resilience was associated with lower reports of anger, but its protective effect diminished over time. First- and second-time deployers reporting low resilience displayed different anger trajectories (stable *v*. decreasing, respectively).

**Conclusions:**

Change in anger from pre- to post-deployment varies based on pre-deployment factors. The observed differences in anger trajectories suggest that efforts to detect and reduce problematic anger should be tailored for first-time *v*. repeat deployers. Ongoing screening is needed even for soldiers reporting high resilience before deployment, as the protective effect of pre-deployment resilience on anger erodes over time.

## Introduction

Studies of Iraq and Afghanistan veterans have identified a range of behavioral health problems that appear related to combat deployments (Bryan et al., 2015; Hoge et al., 2004; Hoge, Auchterlonie, & Milliken, 2006; Smith et al., 2008). To prevent outcomes such as posttraumatic stress disorder (PTSD) and suicide, robust research programs have emerged to understand their phenomenology and help lessen their impact within military populations (e.g. National Center for PTSD, 2020; Ursano et al., 2014). However, combat deployments also may impact other, under-recognized outcomes that carry significant costs for service members.

Anger problems have been relatively understudied, yet available evidence suggests they are common and concerning to combat-deployed service members. In a nationally-representative sample of Iraq and Afghanistan veterans receiving Veterans Affairs medical care, 57% identified difficulty controlling anger as a problem experienced since homecoming, making it the most commonly-reported reintegration problem (Sayer et al., 2010). A survey of National Guard and Reserve members similarly found that 53% reported anger problems (Worthen et al., 2014). Additionally, data from the Millennium Cohort Study indicate that 17% of current and former service members screened positive for ‘problematic anger’, or intense anger that is likely to result in significant distress or impairment (Adler, LeardMann, Roenfeldt, Jacobson, & Forbes, 2020). Anger problems are in turn linked to adverse outcomes such as interpersonal violence (Birkley & Eckhardt, 2015; Park, Sullivan, Riviere, Merrill, & Clarke-Walper, 2021), workplace aggression (Hershcovis et al., 2007), legal problems (Elbogen et al., 2012), risky behaviors (Adler, Britt, Castro, McGurk, & Bliese, 2011), and mental health conditions such as PTSD, depression, substance abuse, and suicidality (Dillon et al., 2020; Gonzalez, Novaco, Reger, & Gahm, 2016; Rona et al., 2015; Wilk, Quartana, Clarke-Walper, Kok, & Riviere, 2015; Worthen et al., 2015). Given the impact of problematic anger on service members, further investigation is needed to identify factors that influence this outcome.

Combat deployments may be a risk factor for problematic anger (Adler et al., 2020; Gallaway, Fink, Millikan, & Bell, 2012; MacManus et al., 2015; Rona et al., 2015), but the nature of this relationship is poorly understood. A key question centers on how anger changes over the deployment cycle. Studies have examined anger-related outcomes (e.g. aggression, violent crime) during combat deployments (Rosellini et al., 2018) and after returning from deployment (Bliese, Wright, Adler, Thomas, & Hoge, 2007; Cabrera, Adler, & Bliese, 2016; Gallaway et al., 2012). However, these studies have not documented if or how anger changes from pre- to post-deployment. Moreover, information is lacking regarding factors that impact change in anger across the deployment cycle, and hence which soldiers may be at elevated risk for developing anger problems during this period.

Previous experience with combat deployment is an important factor to consider in efforts to identify vulnerable soldiers. Evidence suggests that prior deployment experience modifies the risk of some, but not all, post-deployment mental health outcomes (Boulos & Zamorski, 2013; Fear et al., 2010; Maupin, Tvaryanas, White, & Lysfjord, 2017; Reger, Gahm, Swanson, & Duma, 2009; Williams et al., 2015). Information regarding the impact of first-time combat deployment on anger, and whether multiple deployments have a cumulative or other distinctive effect on anger, would be valuable to the military. Research should also prioritize identifying risk or protective factors that have clear implications for how anger problems might be prevented. For example, evidence suggests that high self-reported resilience is associated with reduced risk of mental health problems in service members (Bezdjian, Schneider, Burchett, Baker, & Garb, 2017; Campbell-Sills et al., 2018; Hoopsick et al., 2021). If resilience buffers the effects of combat deployment on anger, programs to improve resiliency skills (e.g. Bliese, Adler, & Castro, 2011a; Brunwasser, Gillham, & Kim, 2009; Jha et al., 2015) could be applied in an attempt to reduce the incidence of anger problems in soldiers.

In this study, we add to existing research on problematic anger in service members by examining trajectories of anger over the course of a combat deployment. We examine a large cohort of soldiers who participated in a longitudinal study that included a pre-deployment baseline and post-deployment follow-ups reflecting acute and longer-term readjustment periods (3 and 9 months post-deployment, respectively). Using this longitudinal design, we investigate changes in anger over the course of deployment to Afghanistan and subsequent re-deployment to the USA. We also examine the main and interactive effects of prior deployment experience and pre-deployment perceptions of resilience on overall levels and change in anger over the course of a combat deployment. Finally, to evaluate the extent to which inclusion of higher-risk soldiers impacts the results of our models, we perform a sensitivity analysis restricting the sample to soldiers with no pre-deployment lifetime mental disorders.

## Methods

### Study overview/participants

The Pre/Post Deployment Study (PPDS) of the Army Study to Assess Risk and Resilience in Servicemembers (Army STARRS; Kessler et al., 2013a; Ursano et al., 2014) is a prospective panel survey of three US Army Brigade Combat Teams (BCTs) that deployed to Afghanistan in 2012 for an average of 10 months. Baseline (T0) evaluation occurred 1–2 months before deployment. Follow-ups were conducted approximately 1 month (T1), 3 months (T2), and 9 months (T3) post-deployment. Written informed consent was obtained for survey participation and for linking survey responses to US Army/Department of Defense administrative records. Procedures were approved by the Human Subjects Committees of the collaborating institutions.

At T0, 9949 soldiers were present for duty in the three BCTs, 9488 (95.3%) consented to participate in the survey, and 8776 (88.2%) also consented to linking survey responses and administrative records. Of the soldiers who consented to both the T0 survey and administrative data linkage, 7741 subsequently deployed to Afghanistan and were eligible for inclusion in this study. Because the analysis examines change in anger from pre- to post-deployment, the eligible sample was restricted to those who completed the T0 survey and either the T2 follow-up only (*n* = 26), the T3 follow-up only (*n* = 958), or both T2 and T3 follow-ups (*n* = 6354). Forty soldiers were excluded due to missing deployment history data, yielding a final sample of 7298 soldiers.

### Measures

#### Problematic anger

A measure of problematic anger was created for the study based on four items adapted from the Composite International Diagnostic Interview Screening Scales (CIDI-SC; Kessler & Ustun, 2004). The items were administered at T0, T2, and T3 and inquired, ‘How often do you feel…(1) irritated, annoyed, or grouchy; (2) so angry that you think you might explode; (3) a lot more angry than most people would be in the same situation; and (4) that your anger is out of control’. Respondents rated the frequency of these feelings on a five-point scale from ‘none of the time’ (coded ‘1’) to ‘all or almost all of the time’ (coded ‘5’). In the T0 survey, the items were not anchored to a specific time-frame; in the T2 and T3 surveys, they were anchored to the past 30 days. Factor analyses suggested the four items loaded on a single factor (item-factor loadings = 0.73–0.89), and the items demonstrated good-to-excellent internal consistency at each wave (average Cronbach's *α* = 0.90). A problematic anger score was derived by averaging the ratings of the four items with higher scores reflecting more frequent anger.

#### Combat deployment experience

Prior combat deployment experience was assessed using a T0 item inquiring how many times the respondent had received a combat zone tax exclusion during his or her Army career. Soldiers were categorized as having 0, 1, or 2+ prior combat deployments.

#### Resilience

A prior report (Campbell-Sills et al., 2018) describes the STARRS resilience scale, which was administered at T0. The scale was prefaced by, ‘How would you rate your ability to handle stress in each of the following ways?’ Soldiers rated their abilities to ‘keep calm and think of the right thing to do in a crisis’, ‘manage stress’, ‘try new approaches if old ones don't work’, ‘get along with people when you have to’, and ‘keep your sense of humor in tense situations’ as poor, fair, good, very good, or excellent (coded ‘1’ to ‘5’). Ratings of the five items had good internal consistency in this sample (Cronbach's *α* = 0.89), and were averaged to create a total resilience score with higher scores reflecting greater perceived resilience.

#### Covariates

Socio-demographic characteristics were assessed in the T0 survey and included as covariates in all models. Socio-demographic variables included age, sex, race (white, under-represented minority), education (high-school, college, graduate school), and marital status (married, single, other). Given that anger problems are linked to a range of mental health conditions (e.g. Rona et al., 2015), it is also important to account for variance in participant reports of anger attributable to mental disorders. Mental disorders were evaluated in the T0 survey using items adapted from the CIDI-SC (Kessler & Ustun, 2004) and PTSD Checklist-Civilian Version (Weathers, Litz, Herman, Huska, & Keane, 1993). The disorders assessed were lifetime major depressive disorder, mania/hypomania, generalized anxiety disorder, PTSD, panic disorder, intermittent explosive disorder, substance use disorder, oppositional defiant disorder, conduct disorder, and past-6-month attention deficit-hyperactivity disorder. Army STARRS survey-based diagnoses were validated against structured clinical interviews (Kessler et al., 2013b). We derived a composite diagnostic variable reflecting any lifetime mental disorder at T0 (i.e. prior to the index deployment), which was considered present if the respondent met the criteria for any of the aforementioned disorders. This composite lifetime mental disorder variable was included as a covariate in all models.

### Data analysis

The three waves of data were analyzed with mixed-effect growth models (Bliese & Ployhart, 2002; Singer & Willett, 2003) using the *nlme* package in R (Pinheiro, Bates, DebRoy, & Sarkar, 2019). Following the procedure outlined by Bliese and Ployhart (2002), we first calculated an interclass correlation (ICC) to assess the amount of variance in problematic anger associated with individuals (between variance) *v*. repeated measures (within variance). Second, we entered a numeric vector representing each measurement occasion indexed to the number of months since the pre-deployment assessment (TIME) to estimate the monthly linear trajectory of anger. We used the vector (0, 13, 19), which corresponds to baseline, 3 months after the 10-month deployment, and 9 months post-deployment. Parameter estimates for TIME represent the estimated monthly change in anger (representing the trajectory across the entire study period). Third, we tested if allowing unique time-related trajectories improved model fit by estimating models with random TIME slopes across individuals. Results from this step determine whether time-related linear effects found in step 2 generalize to all participants *v*. whether anger trajectories differ at person-specific rates. Finally, we tested for residual autocorrelation as this may impact parameter and standard error estimates. In the models, anger scores were standardized with a mean of 0 and a standard deviation of 1. Thus, scores convey soldiers' self-reported anger relative to the normative level for the study sample.

After ensuring proper model specification using the steps above, a series of models evaluated the main and interactive effects of prior combat deployment experience and perceived resilience on problematic anger, adjusting for socio-demographic variables and pre-deployment lifetime mental disorder. A sensitivity analysis was subsequently performed within a subsample of soldiers with no pre-deployment lifetime mental disorders. This allowed us to evaluate if the findings of the main analysis were substantially impacted by the inclusion of higher-risk soldiers, and provided a direct assessment of the effects of combat deployment on problematic anger in soldiers who would be characterized as lower risk.

## Results

Socio-demographic and service characteristics of the study sample are presented in Table 1 and correlations among continuous study measures are shown in online Supplementary Table S1. The first step in model building returned an ICC of 0.41, indicating that 41% of the variance in problematic anger was attributable to between-individual factors with the remaining 59% of the variance attributable to variability in repeated measures of anger. The second step established a significant linear trajectory between TIME and anger (TIME = 0.01, s.e. = 0.00, *t* value = 7.51, *p* < 0.01). The third step tested whether trajectories differed significantly across individuals. Results indicated that the model allowing for random slopes provided a better fit to the data than a model where linear trajectories were held constant (Log-likelihood ratio test = 190.17, *p* < 0.01). Model fit was not improved by adjusting for residual autocorrelation (Log-likelihood ratio test = 0.96, *ns*).

**Table 1.** Socio-demographic and service characteristics of the study sample (*N* = 7298)

Demographic variable	*N*	Sample frequency (%)
Gender		
Male	6860	94
Female	438	6
Race		
White	4744	65
Non-White	2554	35
Age (mean and s.d.)	Mean = 25.99	s.d. = 6.03
Pre-deployment mental disorder		
Pre-deployment mental disorder	3087	42
No pre-deployment mental disorder	4211	58
Education		
High school	5400	74
College	1752	24
Graduate school	146	2
Marital status		
Married	3868	53
Never married	2773	38
Other	657	9
Prior combat deployment history		
First-time deployment	3795	52
1 Prior deployment	1751	24
2+ Prior deployments	1752	24

Based on these results, we used the random slope model as the baseline model to identify the predictors of differences in anger trajectories. Table 2 presents the results of three increasingly complex mixed-effect growth models to assess the effects of between-individual factors on anger trajectories. Model 1 estimates the effects of between-individual factors on the average level of problematic anger over the course of the study. Model 2 simultaneously estimates the effects of prior combat deployment history and perceived resilience on trajectories of anger (TIME × Deployment History; TIME × Resilience). Model 3 estimates the moderating effect of pre-deployment resilience on the relationship between deployment history and anger trajectories (TIME × Deployment History × Resilience).

**Table 2.** Mixed-effect growth models of problematic anger among combat-deployed soldiers (*N* = 7298)

	Model 1		Model 2		Model 3	
	Est	s.e.	Est	s.e.	Est	s.e.
Intercept	0.88**	0.07	0.90**	0.08	0.89**	0.08
Male^a^	0.02	0.03	0.02	0.03	0.01	0.03
Age	−0.01**	0.00	−0.01**	0.00	−0.01**	0.00
Non-White^b^	0.01	0.02	0.01	0.02	0.01	0.02
Education						
College *v*. high school	−0.05*	0.20	−0.04*	0.02	−0.04*	0.02
Graduate school *v*. high school	−0.07	0.06	−0.06	0.05	−0.06	0.05
Marital status						
Single *v*. married	−0.08**	0.02	−0.08**	0.02	−0.08**	0.02
Other *v*. married	0.04	0.03	0.03	0.03	0.03	0.03
Pre-deployment mental disorder^c^	0.68**	0.02	0.68**	0.02	0.68**	0.02
TIME	0.01**	0.00	−0.04**	0.00	−0.04**	0.01
Prior combat deployment history						
1 deployment *v*. 0 deployments	0.12**	0.02	0.48**	0.09	0.56**	0.10
2+ deployments *v*. 0 deployments	0.19**	0.02	0.67**	0.09	0.63**	0.10
Resilience	−0.25**	0.01	−0.26**	0.01	−0.26**	0.01
TIME × 1 prior deployment			−0.01**	0.00	−0.03*	0.01
TIME × 2+ prior deployments			−0.01**	0.00	0.00	0.01
TIME × resilience			0.01**	0.00	0.01**	0.00
1 Prior deployment × resilience			−0.09**	0.02	−0.11**	0.03
2+ Prior deployments × resilience			−0.11**	0.02	−0.10**	0.03
TIME × 1 prior deployment × resilience					0.01*	0.00
TIME × 2+ prior deployments × resilience					0.00	0.00
Variance components						
Intercept	0.14	0.38	0.15	0.38	0.15	0.38
TIME	0.00	0.03	0.00	0.03	0.00	0.03
Residual	0.50	0.70	0.49	0.70	0.49	0.70
Log-likelihood^d^	−21 203.32		−21 092.46		−21 088.81	
−2LLR^d^			221.73**		7.30*	

*Note*. Anger is standardized.

a Male is dummy coded (0 = Female, 1 = Male).

b Non-White was dummy coded (0 = White, 1 = Non-White).

c Pre-deployment mental disorder was dummy coded (0 = No reported mental disorder prior to T0; 1 = One or more reported mental disorders prior to T0).

d Log-likelihood values calculated from models using maximum likelihood to allow for −2 Log-likelihood ratio test across models with differing fixed-effects (Pinheiro & Bates, 1999).

Model 1 indicates that, in general, problematic anger scores increased by 0.01 per month, representing a trajectory that leads to a 0.19 standard deviation increase by the end of the study. This overall increase, while small, is statistically significant. Recall, however, that model fit was improved by allowing TIME slopes to randomly vary, suggesting the need to consider individual predictors of anger trajectories. Model 1 also reveals that both combat deployment history and perceived resilience at baseline predict average levels of problematic anger. Soldiers with either one (0.12, *p* < 0.01) or 2+ (0.19, *p* < 0.01) prior deployments report higher anger overall (i.e. averaged across all time points) than first-time deployers. Not shown in the table is that soldiers with one prior deployment also report significantly lower average anger than soldiers with 2+ prior deployments (−0.07, s.e. = 0.02, *p* < 0.01). Finally, model 1 results indicate that higher self-reported resilience at baseline is associated with lower anger overall (−0.25, *p* < 0.01).

**Fig. 1.**
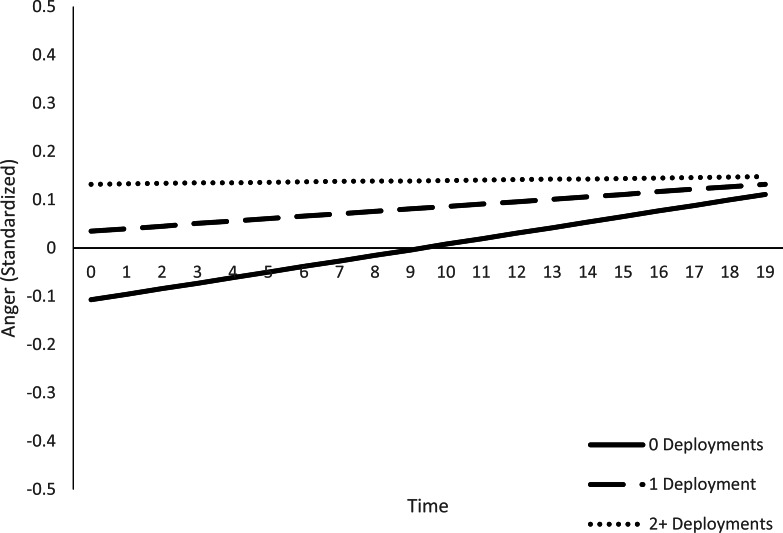
Predicted values of anger trajectories by prior combat deployment history.

Model 2 suggests anger trajectories were significantly influenced by both prior combat deployment experience and self-reported resilience. Soldiers with one (−0.01, *p* < 0.01) and 2+ (−0.01, *p* < 0.01) prior deployments reported less growth in anger over time than first-time deployers. The form of the interaction involving TIME and deployment history is presented in Fig. 1. Soldiers with 2+ prior deployments reported higher initial levels of anger, but smaller increases in anger over time. In contrast, both first-time deployers and soldiers with one prior deployment reported lower anger at baseline, but larger increases in anger over time.

**Fig. 2.**
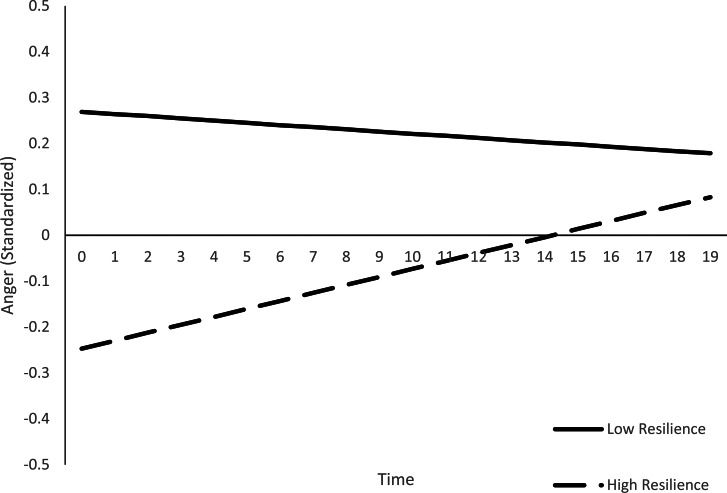
Predicted values of anger trajectories by perceived resilience. Low resilience is defined as 1 s.d. below the mean and high resilience is defined as 1 s.d. above the mean resilience score for the sample.

Model 2 also suggests that higher self-reported resilience was related to more growth in anger over time (0.01, *p* < 0.01). Figure 2 visualizes the two-way interaction involving TIME and resilience. Soldiers who reported high resilience tended to report low baseline anger, whereas those who reported low resilience typically reported high baseline anger. Over time, both groups regressed toward the mean, i.e. anger increased over time among those reporting high resilience and decreased over time in those reporting low resilience. The form of the interaction suggests that the strength of the protective effect of baseline resilience on problematic anger diminishes over time. Despite the waning of the protective effect, Fig. 2 indicates that the levels of anger remained lower at 9 months post-deployment in those with high *v*. low resilience.

**Fig. 3.**
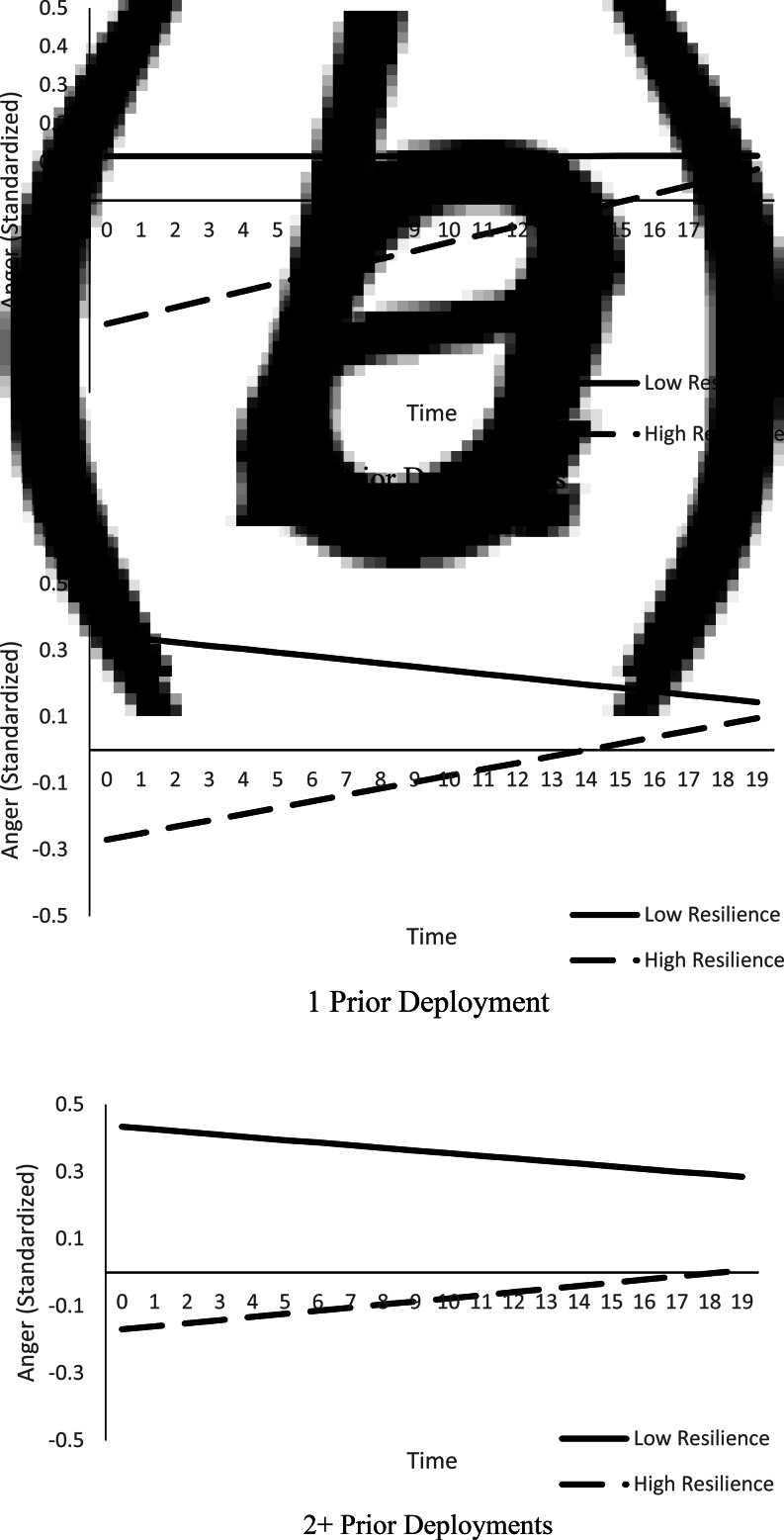
Moderating effect of resilience on anger trajectories, per prior deployment history category. Panels *a*, *b*, and *c* show results for soldiers with 0, 1, and 2+ prior combat deployments, respectively. In all panels, resilience is defined as 1 s.d. below the mean and high resilience is defined as 1 s.d. above the mean resilience score for the full sample.

Model 3 includes the three-way interaction between TIME, deployment history, and self-reported resilience, which tests whether baseline resilience modifies anger growth equally across deployment history conditions. The estimate for the three-way interaction term involving the contrast between those with one previous deployment and those with no previous deployments was significant (0.01, *p* < 0.01). The first two panels of Fig. 3 illustrate the form of this three-way interaction. The effect of resilience on anger trajectories among first-time deployers (Fig. 3*a*) and those with one prior deployment (Fig. 3*b*) generally follows the same pattern observed in Fig. 2, suggesting that baseline resilience becomes less strongly related to reports of anger over time. Figure 3*a* shows that among first-time deployers, this two-way interaction is muted because the low resilience group maintains relatively stable anger. In contrast, Fig. 3*b* shows that among those with one previous deployment, baseline reports of low resilience are associated with higher problematic anger, but over time anger levels regress toward the mean. Figures 3*a* and 3*b* further show that the increase in anger among first-time *v*. second-time deployers who reported high resilience at baseline appears similar. Figure 3*c* shows the change in anger among soldiers with 2+ prior deployments who reported low *v*. high resilience. However, as Table 2 indicates, the contrast between those with 2+ prior deployments and no prior deployments was non-significant.

### Sensitivity analysis

The sensitivity analysis evaluated the extent to which the main results were influenced by the inclusion of higher-risk soldiers by restricting the analysis sample to soldiers with no pre-deployment lifetime mental disorders (*n* = 4211; online Supplementary Table S2). Results were similar to the main analysis in that (1) anger increased from pre- to 9 months post-deployment in the subsample overall; (2) more combat deployment experience was associated with higher average anger, while increases in anger over time were most pronounced among first-time deployers (online Supplementary Fig. S1); and (3) higher pre-deployment resilience was associated with lower average anger, but more growth in anger over time (online Supplementary Fig. S2). A difference was the TIME × Deployment History × Resilience interaction was non-significant in this subsample. Additionally, the models predicted increasing anger trajectories for soldiers reporting both high and low resilience before deployment. Although the increase was less pronounced among those reporting low baseline resilience, the pattern contrasts with the modest decreasing anger trajectory predicted for the low resilience group from the full sample. However, in both cases, these patterns (decreasing trajectory in the main analysis *v*. a smaller increase in the sensitivity analysis) are likely related to the fact that anger levels are already substantially elevated before deployment in soldiers reporting low resilience.

## Discussion

In a cohort of more than 7000 US Army soldiers, we found evidence of significant heterogeneity of anger trajectories over the course of combat deployment and post-deployment readjustment. When reports of problematic anger were considered in the aggregate, a small increase over time was observed, broadly converging with prior evidence suggesting a link between combat deployment and increases in anger problems (Adler et al., 2020; MacManus et al., 2015). However, the modest average increase in anger did not represent the change patterns for many individual soldiers, as model fit was improved substantially by allowing individuals to have person-specific trajectories. Furthermore, differences in anger trajectories were related to pre-deployment factors such as prior deployment experience and perceived resilience.

Prior combat deployment experience was associated with both the overall level and pattern of change in problematic anger from pre- to 9 months post-deployment. The relationship between prior combat deployments and overall anger (i.e. averaged across time) approximated a dose–response relationship, wherein soldiers with no prior deployments reported the lowest levels of anger, those with one prior deployment reported intermediate levels of anger, and those with multiple prior deployments reported the highest levels of anger. The effect of prior combat deployment on anger trajectories further demonstrated that soldiers with no prior deployments reported the greatest increases in anger over time. Those with one prior deployment experienced more moderate increases in anger, while anger remained relatively high and stable across time in those with multiple prior deployments. These effects were observed controlling for potential confounds of deployment experience (e.g. age), and were replicated in a sensitivity analysis that included only soldiers without pre-deployment mental disorders.

We also examined the effects of perceived resilience at baseline. Identifying as highly resilient before deployment was associated with lower anger overall (i.e. averaged across time), but did not protect against experiencing an increase in anger over time. On the contrary, soldiers reporting high resilience exhibited low levels of baseline anger that increased over time, whereas those reporting low resilience displayed high levels of baseline anger that decreased over time. These effects were largely replicated in the subgroup with no pre-deployment mental disorders. Thus, while perceiving oneself as resilient may indicate less susceptibility to anger in general, our results do not justify targeting perceptions of resilience as a strategy to prevent increases in anger from pre- to post-deployment.

In terms of understanding why the protective effect of self-reported resilience was robust at baseline yet waned over time, a possible explanation is that the pre-deployment period constitutes a highly demanding time when a soldier's sense of resilience relates strongly to his or her levels of anger. As the experience of the stressor becomes more distant (i.e. as time elapses after return from deployment), the relationship between perceived resilience and anger may weaken, resulting in regression to the mean among soldiers reporting both high and low resilience. An alternative possibility, though more speculative, is that overestimating one's own resilience before or during stress exposure has detrimental effects (i.e. increases in anger when one does not cope as well as anticipated), whereas underestimating one's resilience has favorable effects due to the psychological benefit of coping better than expected (cf. situational benefits of ‘defensive pessimism’; Norem & Chang, 2002). Overall, the results highlight that it is challenging to understand how factors like resilience relate to outcomes over time if the first measurement occasion is during a time of significant stress.

The analysis also considered whether the effect of resilience on anger trajectories varied based on previous combat deployment experience. Results suggested that the protective effect of high resilience subsided over time, regardless of whether soldiers had previously deployed or were on their first deployment. Instead, the interactive effect of deployment history and resilience was related to a lack of change in anger over time in first-time deployers who reported low resilience, which contrasted with a pattern of decreasing anger over time in second-time deployers who reported low resilience. Second-time (*v*. first-time) deployers with low self-reported resilience endorsed substantially higher levels of anger before deployment, which may be attributable to having knowledge of the challenges to come (from having deployed before) and feeling unprepared to cope effectively. Anger related to the anticipation of unmanageable stress may subside as the challenges are endured, resulting in decreasing anger trajectories. On the other hand, first-time deployers with low self-reported resilience may simply experience a level of anger that is normative for them during the pre-deployment period (not having a clear idea of challenges to come), which is less subject to change over time.

These results contribute to the literature on combat deployment and problematic anger by assessing post-deployment anger in relation to a pre-deployment baseline, examining how prior deployment experience affects overall levels and growth of anger over time, and investigating the effects of a modifiable protective factor. The findings show that repeat deployers are more likely to report elevated anger before deployment, whereas first-time deployers are vulnerable to larger increases in anger from pre- to post-deployment. Possible explanations for the higher overall levels of anger in repeat deployers include selection factors (e.g. differences between individuals who continue service and deploy multiple times *v*. those who separate after a first deployment) as well as potential causal links between the experience of deploying multiple times and problematic anger. For instance, stress sensitization resulting from more exposures to deployment stressors (Bliese, Thomas, McGurk, McBride, & Castro, 2011b; Smid, Kleber, Rademaker, van Zuiden, & Vermetten, 2013) could lower the threshold for problematic anger among repeat deployers, who might then display more chronically elevated anger. Additionally, anger may be more acceptable than other negative emotions in military settings (Adler, Brossart, & Toblin, 2017; Adler, Wright, Bliese, Eckford, & Hoge, 2008), or facilitate performance in certain contexts (Geddes & Callister, 2007; Naifeh, Gonzalez, Herberman Mash, Fullerton, & Ursano, 2021). In experienced soldiers preparing to deploy, elevated anger could reflect a mindset focused on overcoming threats to self, unit, or mission. Yet reports of anger remained relatively high through 9 months post-deployment in the group with multiple prior deployments, suggesting that anger tends to persist beyond the period where it may have utility from a combat readiness perspective.

Prior research indicates that even anger that is deemed ‘useful’ is associated with distress and impairment in service members (Adler et al., 2017), and that anger can cross a threshold that leads to impaired performance (Geddes & Callister, 2007). Thus, a key question is how to prevent escalating anger trajectories that result in markedly elevated anger. The first step is detection; to this end, validated screens for problematic anger could be incorporated into routine health monitoring (e.g. Pre/Post Deployment or Periodic Health Assessments). Such screening could help identify soldiers who might benefit from interventions to decrease anger, its precipitants (e.g. stress), or co-occurring problems (e.g. relationship problems, PTSD, suicidal thoughts/behaviors).

While more research is needed to guide interventions, one possibility is that education about anger – e.g. as a reaction to stress and potential signal of other problems – could be useful in raising soldiers' awareness of its detrimental effects and increasing the likelihood that they would report problematic anger to treatment providers. Such interventions could be tailored for first-time *v*. repeat combat deployers, given the distinct patterns of anger observed in these groups. For example, repeat deployers may benefit from outreach before deployment (when anger may already be elevated), whereas first-time deployers might be most usefully targeted during or shortly after deployment (for intervention focused on recognizing and addressing increases in anger). When anger problems are detected via routine screening or disclosure to clinicians/unit leaders, or through observable behavior/disciplinary problems, soldiers may be referred to anger management programs. Available evidence suggests that combat-deployed soldiers are receptive to these programs (Judkins & Bradley, 2017), and that participants report reductions in anger and improvements in interpersonal functioning (Kalkstein, Scott, Vickers Smith, & Cruz, 2018; Linkh & Sonnek, 2003; Shea, Lambert, & Reddy, 2013).

The resilience findings were complex but did indicate that high perceived resilience was associated with lower anger overall. However, high resilience did not predict adaptive (e.g. low stable) anger trajectories or mitigate increases in anger within vulnerable groups (e.g. first-time deployers). The increases in anger seen among soldiers who identified as highly resilient, as well as in those without pre-existing mental disorders, suggest that ongoing screening is needed even for soldiers presumed to be ‘low-risk’ for experiencing problematic anger following combat deployments. The finding that the effect of baseline resilience on anger diminished over time is perhaps not surprising, given that resilience is considered to be a dynamic process (Southwick, Bonanno, Masten, Panter-Brick, & Yehuda, 2014) and could be impacted by the experiences of deploying and readjusting to life in the USA. Additionally, perceptions of resilience may vary depending on the type of stressor that is anticipated. Our measure of resilience prompted soldiers to consider their capacity to manage stress in general, not their ability to manage stress resulting from deployment. Future studies should examine changes in perceived resilience over the course of combat deployment, and evaluate whether these vary systematically in relation to anger or other problems. Such inquiry may help determine whether resiliency training has a role to play in preventing problematic anger in soldiers. It may also be informative to assess soldiers' perceptions of their abilities to cope with deployment experiences specifically, and to evaluate how these relate to post-deployment mental health.

Study limitations included reliance on self-report measures, which are vulnerable to response bias. Second, the anger measure was developed for this study and has not been validated. That is, we do not know how scores on the measure relate to occupational, interpersonal, or legal/disciplinary problems. Consequently, we were unable to apply established cut-scores to identify soldiers with levels of anger that are likely to lead to dysfunction. Third, anger is associated with a range of internalizing and externalizing symptoms (e.g. Rona et al., 2015), and we chose to capture variance in anger related to pre-deployment mental health by adjusting for the presence of any lifetime mental disorder. It was beyond the scope of the study to investigate possible differential associations of mental disorders with anger across the deployment cycle; however, future investigations may find value in attempting to clarify relationships between specific mental disorders (e.g. PTSD, substance use disorders) and deployment-related anger trajectories. Finally, results may not generalize to women (who comprised only 6% of the participating BCTs), Reserve/Guard personnel, or members of other branches of the military.

## Conclusion

Substantial variation exists in the anger trajectories of combat-deployed soldiers, and patterns of change appear related to prior deployment experience and pre-deployment perceptions of resilience. Repeat deployers are more likely to exhibit elevated anger before deployment, whereas first-time deployers are vulnerable to greater increases in anger from pre- to post-deployment. Therefore, the optimal timing for interventions to prevent or reduce problematic anger may differ based on soldiers' deployment histories. Our findings also suggest that high pre-deployment resilience may protect soldiers from problematic anger, but that this effect erodes over time. Thus, ongoing screening for problematic anger is needed even for soldiers who appear low-risk before deployment. Overall, the study reveals complex relationships between prior deployment experience, perceived resilience, and problematic anger; and highlights the need for more research to identify factors that may mitigate the risk of maladaptive anger trajectories in combat-deployed soldiers.

## Supplementary material

For supplementary material accompanying this paper visit https://doi.org/10.1017/S0033291721003779. click here to view supplementary material
