# Supplementary material for: Effects of prior deployments and perceived resilience on anger trajectories of combat-deployed soldiers
Source: Psychol Med. 2021 Nov 22;53(5):2031–40. doi: 10.1017/S0033291721003779 (PMC9124235; doi:10.1017/S0033291721003779)
Supplement: Supplementary file 1 [file S0033291721003779sup001.docx]

**Supplementary Material**

**Supplementary Table 1.** Means, standard deviations, Cronbach's alphas, and correlations among continuous study measures

**Supplementary Table 2.** Mixed-effect growth models of problematic anger in a subsample of soldiers with no pre-deployment history of mental disorders (n=4211)

**Supplementary Figure 1.** Predicted values of anger trajectories by prior combat deployment history among soldiers with no pre-deployment history of mental disorders

**Supplementary Figure 2.** Predicted values of anger trajectories by resilience among soldiers with no pre-deployment history of mental disorders

**Supplementary Table 1.** Means, standard deviations, Cronbach's alphas, and correlations among continuous study measures

|  | **Mean** | **SD** | 1 | 2 | 3 | 4 |
| --- | --- | --- | --- | --- | --- | --- |
| 1. Resilience (T0) | 3.99 | .84 | (.89) |  |  |  |
| 1. Anger (T0) | 1.70 | .79 | -.34 | (.88) |  |  |
| 1. Anger (T2) | 1.76 | .88 | -.16 | .41 | (.89) |  |
| 1. Anger (T3) | 1.85 | .97 | -.15 | .36 | .50 | (.92) |

*Note.* Cronbach's Alpha reported on diagonal

**Supplementary Table 2**

Mixed-effect growth models of problematic anger in a subsample of soldiers with no pre-deployment history of mental disorders (n=4211)

|  | **Model 1** | | **Model 2** | | **Model 3** | |
| --- | --- | --- | --- | --- | --- | --- |
|  | *Est* | *SE* | *Est* | *SE* | *Est* | *SE* |
| Intercept | 0.91** | 0.09 | 0.91** | 0.10 | 0.90** | 0.11 |
| Male^a^ | 0.04 | 0.04 | 0.03 | 0.04 | 0.03 | 0.04 |
| Age | -0.01** | 0.00 | -0.01** | 0.00 | -0.01** | 0.00 |
| Non-White^b^ | -0.04* | 0.02 | -0.05* | 0.02 | -0.05* | 0.02 |
| Education |  |  |  |  |  |  |
| College vs. High School | 0.03 | 0.03 | 0.03 | 0.03 | 0.03 | 0.03 |
| Graduate School vs. High School | 0.02 | 0.07 | 0.03 | 0.07 | 0.03 | 0.07 |
| Marital Status |  |  |  |  |  |  |
| Single vs. Married | -0.03 | 0.03 | -0.03 | 0.03 | -0.03 | 0.03 |
| Other vs. Married | 0.03 | 0.04 | 0.03 | 0.04 | 0.03 | 0.04 |
|  |  |  |  |  |  |  |
| TIME | 0.02** | 0.00 | -0.01 | 0.01 | -0.01 | 0.01 |
|  |  |  |  |  |  |  |
| Prior Combat Deployment History |  |  |  |  |  |  |
| 1 Deployment vs. 0 Deployments | 0.13** | 0.03 | 0.28 | 0.15 | 0.34* | 0.17 |
| 2+ Deployments vs 0 Deployments | 0.17** | 0.03 | 0.63** | 0.16 | 0.57** | 0.17 |
| Resilience | -0.21** | 0.01 | -0.21** | 0.02 | -0.21** | 0.02 |
|  |  |  |  |  |  |  |
| TIME * 1 Deployment |  |  | -0.01* | 0.00 | 0.03 | 0.02 |
| TIME * 2+ Deployments |  |  | -0.01** | 0.00 | 0.00 | 0.02 |
| TIME * Resilience |  |  | 0.01** | 0.00 | 0.01** | 0.00 |
|  |  |  |  |  |  |  |
| 1 Deployment * Resilience |  |  | -0.03 | 0.04 | -0.05 | 0.04 |
| 2+ Deployments * Resilience |  |  | -0.10** | 0.04 | -0.08* | 0.04 |
|  |  |  |  |  |  |  |
| TIME * 1 Deployment * Resilience |  |  |  |  | 0.00 | 0.00 |
| TIME * 2+ Deployments * Resilience |  |  |  |  | 0.00 | 0.00 |
|  |  |  |  |  |  |  |
| Variance Components |  |  |  |  |  |  |
| Intercept | .04 | .21 | .04 | .21 | .04 | .21 |
| TIME | .00 | .05 | .00 | .05 | .00 | .05 |
| Residual | .53 | .72 | .52 | .72 | .52 | .72 |
|  |  |  |  |  |  |  |
| Loglikelihood^c^ | -12083.11 | | -12061.82 | | -12060.64 | |
| -2LLR^c^ |  |  | 42.57** | | 2.37 | |

*Note*. Anger is standardized. ^a^Male is dummy coded (0=Female, 1=Male). ^b^Non-White was dummy coded (0=White, 1=Non-White). ^c^Loglikelihood values calculated from models using maximum likelihood to allow for -2 Loglikelihood Ratio test across models with differing fixed-effects (Pinheiro & Bates, 1999)

**Supplementary Figure 1.** Predicted values of anger trajectories by prior combat deployment history among soldiers with no pre-deployment history of mental disorders.

**Supplementary Figure 2**. Predicted values of anger trajectories by resilience among soldiers with no pre-deployment history of mental disorders. Low resilience is defined as 1 SD below the mean and high resilience is defined as 1SD above the mean resilience score for the subsample with no pre-deployment mental disorders.
